# Metabolic dysfunction–associated steatotic liver disease with alcohol- and iron overload–related cholestatic liver injury: a case report

**DOI:** 10.3389/fmed.2026.1805756

**Published:** 2026-07-15

**Authors:** Bin Cui, Hao Li, Rihua Cui, Xu Jiang, Xinglin Jin

**Affiliations:** 1 Department of Hepatobiliary Surgery, Affiliated Hospital of Yanbian University, Yanji, China; 2 Department of Medical Insurance, Affiliated Hospital of Yanbian University, Yanji, China

**Keywords:** alcohol-associated liver disease, case report, cholestasis, iron overload, metabolic dysfunction

## Abstract

A 38-year-old woman with a >10-year history of heavy alcohol consumption presented with acute-onset jaundice and massive hepatomegaly. Laboratory tests revealed a cholestatic-pre-dominant liver injury pattern with extreme γ-glutamyl transferase elevation (>1,000 U/L) and marked hyperferritinemia (>1,500 ng/ml). Imaging excluded extrahepatic biliary obstruction. Liver biopsy demonstrated steatohepatitis with ductular reaction and stage F2 fibrosis. Metagenomic next-generation sequencing (mNGS) was negative for infectious pathogens. After alcohol abstinence, metabolic intervention, a short empiric corticosteroid course, and supportive therapy, liver function gradually improved. This case highlights a reversible cholestatic phenotype in alcohol-associated steatotic liver injury with metabolic dysfunction and suspected secondary iron overload.

## 1 Introduction

Metabolic dysfunction-associated steatotic liver disease (MASLD) has recently replaced NAFLD according to international consensus (1, 2).

The concept of MetALD further recognizes the coexistence of metabolic dysfunction and alcohol exposure (4, 5).

This case report was prepared in accordance with the CARE guideline (6).

## 2 Case presentation

### 2.1 Patient information

A 38-year-old Korean woman was admitted with progressive yellowing of the skin, mucosa, and sclera lasting 3 days. She reported mild abdominal distension, nausea, vomiting, and cough, without fever, chills, or significant abdominal pain. Initial empirical anti-infective therapy at a local emergency department was ineffective.

Her medical history included acute glomerulonephritis in early adulthood, hypertension treated with metoprolol, and chronic anemia managed with folic acid. She reported long-term heavy alcohol consumption (>10 years, approximately 500 g of spirits daily). No documented period of abstinence before admission was available. Childhood history included repeated exposure to glucocorticoids and antibiotics. She underwent bilateral total hip arthroplasty for femoral head necrosis in 2018 and cesarean delivery in 2020. She denied diabetes, tuberculosis, coronary heart disease, or relevant family history. Penicillin and cephalosporin allergies were documented.

### 2.2 Clinical findings

On admission, vital signs were stable. Physical examination revealed marked jaundice and massive hepatomegaly extending below the umbilicus toward the pelvic plane. The liver was firm with blunt margins and mild tenderness. No ascites, splenomegaly, abdominal wall varices, or signs of chronic portal hypertension were observed.

### 2.3 Timeline

- Greater than 10 years before admission: heavy alcohol consumption; chronic anemia;
- 2018: bilateral total hip arthroplasty;
- 2020: cesarean section;
- Three days before admission: acute onset of jaundice;
- Hospital Day 1: admission with cholestatic liver injury;
- Hospital Day 2–3: CT and MRCP excluded biliary obstruction;
- Hospital Day 4: liver biopsy performed;
- Hospital Day 5: mNGS negative for infection;
- Hospital Day 6–8: short-course corticosteroid therapy;
- Discharge: alcohol abstinence and metabolic intervention initiated;
- Follow-up (4 weeks): resolution of jaundice and biochemical improvement;

### 2.4 Diagnostic assessment

Laboratory tests showed a cholestatic-pre-dominant pattern with markedly elevated γ-glutamyl transferase (>1,000 U/L), direct hyperbilirubinemia, moderate alkaline phosphatase elevation, and AST pre-dominance over ALT. Inflammatory markers (CRP and IL-6) were elevated without evidence of severe sepsis. Serum ferritin exceeded 1,500 ng/ml, with reduced folate and elevated erythropoietin. Transferrin saturation and MRI-based hepatic iron quantification were not available. Autoimmune liver disease antibodies and viral hepatitis markers were negative.

**Figure 1:**
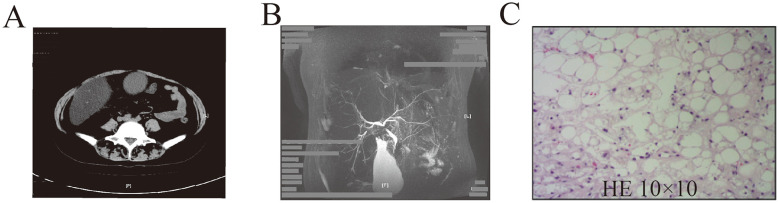
**(A)** Unenhanced abdominal CT (October 17, 2025) shows marked hepatomegaly with diffusely decreased parenchymal density, consistent with fatty liver. No intra- or extrahepatic bile duct dilation or hilar mass is observed. The gallbladder is distended with increased wall density. These findings exclude extrahepatic obstruction and support intrahepatic or functional cholestasis on a metabolic background. **(B)** MRCP (October 18, 2025) demonstrates smooth and patent intra- and extrahepatic bile ducts, common bile duct, and pancreatic duct, with no stricture or stone. The liver is enlarged with periportal (Glisson's sheath) edema, indicating inflammatory or edematous change of the biliary system and small-duct level cholestasis rather than mechanical obstruction. **(C)** Liver biopsy (October 27, 2025; H&E & IHC) reveals diffuse macrovesicular steatosis, focal ballooning, and bile plugs with mild portal inflammation and early fibrosis (approximately F2 stage). Small-duct proliferation and cholangiolar reaction are evident. Immunohistochemistry shows Heppar-1^+^ (hepatocellular origin), CK7^+^ (bile ductular reaction), CD34^+^ (sinusoidal capillarization), and Ki-67^+^ (regenerative activity), confirming steatohepatitis with intrahepatic cholestasis on a metabolic-toxic background.

Computed tomography and magnetic resonance cholangiopancreatography demonstrated diffuse fatty liver, periportal edema, and gallbladder distension without intra- or extrahepatic bile duct dilation. Liver biopsy revealed diffuse steatosis, hepatocyte ballooning, portal inflammation, ductular reaction, and stage F2 fibrosis. Immunohistochemistry confirmed ductular reaction and sinusoidal capillarization. Representative imaging and histopathological findings are shown in Figure 1. Laboratory findings are summarized in Table 1, and serial liver biochemical parameters are presented in Table 2.

**Table 1:** Dynamic changes in blood routine parameters during hospitalization.

Parameter	2025-10-17	2025-10-19	2025-10-20	2025-10-21	2025-10-23
WBC (10^9^/L)	9.73	9.24	12.59	13.54	11.7
RBC (10^12^/L)	2.45	1.99	2.00	2.35	2.37
HGB (g/L)	90	73	73	85	89
%NEUT (%)	82.3	74.4	81.8	80.5	87
%LYMP (%)	8.6	15.4	9.5	9.7	12.4

**Table 2:** Dynamic changes in liver function parameters during hospitalization.

Parameter	2025-10-17	2025-10-19	2025-10-21	2025-10-23	2025-11-14
AST (U/L)	264	250	187	136	167
ALT (U/L)	47	47	39	35	41
ALP (U/L)	159	162	156	173	138
γ-GT (U/L)	1,159	1,287	1,075	1,045	438
TBIL (μmol/L)	172.1	117.3	136.7	133.4	41.0
DBIL (μmol/L)	142.7	101.1	116.8	113.4	32.3
TBA (μmol/L)	92.3	95.4	83.4	56.0	36.6

Metagenomic next-generation sequencing detected no bacterial, viral, fungal, or parasitic pathogens, excluding infectious cholangitis.

- Diagnostic challenges: the combination of jaundice, inflammatory markers, extreme γ-GT elevation, and gallbladder distension closely mimicked atypical cholangitis despite negative imaging, complicating early etiological classification.
- Final diagnosis: alcohol-associated steatohepatitis (F2 fibrosis) with metabolic dysfunction, hyperferritinemia / suspected secondary iron overload, and acute intrahepatic cholestatic liver injury.

### 2.5 Therapeutic intervention

Treatment focused on stabilization, etiological clarification, and risk factor modification. Supportive therapy included ammonia-lowering agents, nutritional support, and correction of electrolyte imbalance. Empirical antibiotics were initiated due to inflammatory markers and allergy history but shortened after negative mNGS results. Hepatoprotective and anti-cholestatic agents were administered. Anemia was corrected with red blood cell transfusion.

Given the markedly elevated inflammatory markers and progressive cholestatic liver injury, and after multidisciplinary team discussion following exclusion of extrahepatic obstruction, infection, and autoimmune liver disease, a short empiric course of dexamethasone (10 mg intravenously once daily for 3 days) was administered as adjunctive therapy. After discharge, strict alcohol abstinence and metabolic intervention, including weight management, metformin, and an SGLT2 inhibitor, were prescribed.

### 2.6 Follow-up and outcomes

Liver function and inflammatory markers improved during hospitalization. At 4-week follow-up, jaundice had resolved, bilirubin and ferritin levels had markedly decreased, and liver enzymes stabilized. No documented period of abstinence before admission was available, but adherence to alcohol abstinence and metabolic therapy after discharge was confirmed through outpatient follow-up and patient self-report. All treatments were well tolerated, and no adverse or unanticipated events were observed.

## 3 Discussion

This case illustrates a mixed-etiology alcohol-associated steatotic liver injury phenotype amplified by metabolic dysfunction and suspected secondary iron overload, presenting as acute intrahepatic cholestasis without mechanical obstruction (3). Because the reported alcohol exposure exceeded the usual MetALD range, alcohol-associated liver disease is likely the dominant driver, with metabolic and iron-related factors contributing to the cholestatic phenotype. Iron-mediated oxidative stress and ferroptosis likely contributed to hepatocellular and ductular injury, helping explain the disproportionate γ-GT elevation (7–13). The integration of liver biopsy and mNGS was critical in excluding infection and guiding targeted therapy. The principal strength lies in the structured, multimodal diagnostic approach. Limitations include the absence of quantitative hepatic iron measurement by MRI-T2^*^, lack of transferrin saturation data, and lack of genetic testing for iron metabolism disorders. Alcohol-associated liver disease with concurrent metabolic dysfunction and iron dysregulation can mimic obstructive cholestasis; accurate diagnosis requires moving beyond exclusion-based reasoning toward mechanism-driven assessment and intervention.

## 4 Patient perspective

The patient reported significant anxiety at disease onset due to fear of irreversible liver damage. She expressed relief after receiving a clear diagnosis and understanding that the condition was reversible. Improvement in jaundice reinforced her motivation to abstain from alcohol and adhere to metabolic therapy.

## 5 Informed consent

Written informed consent was obtained from the patient for publication of this case report and accompanying images.

## 6 Conclusion

In MASLD, concurrent alcohol and functional iron overload can produce reversible intrahepatic cholestasis mimicking obstruction. Extreme hepatomegaly with periportal edema should raise suspicion for metabolic–toxic injury. Integrating molecular testing, imaging, and structured reasoning allows accurate diagnosis and timely management.

## Data Availability

The original contributions presented in the study are included in the article/supplementary material, further inquiries can be directed to the corresponding author.

## References

[1] Eslam M Newsome PN Sarin SK Anstee QM Targher G Romero-Gomez M . A new definition for metabolic dysfunction-associated fatty liver disease: an international expert consensus statement. J Hepatol. (2020) 73:202–9. doi: 10.1016/j.jhep.2020.07.045 32278004

[2] Rinella ME Lazarus JV Ratziu V Francque SM Sanyal AJ Kanwal F . A multisociety delphi consensus statement on new fatty liver disease nomenclature. J Hepatol. (2023) 79:1542–56. doi: 10.1097/HEP.0000000000000696 37364790

[3] Tilg H Moschen AR. Evolution of inflammation in nonalcoholic fatty liver disease: the multiple parallel hits hypothesis. Hepatology. (2010) 52:1836–46. doi: 10.1002/hep.24001 21038418

[4] Gratacós-Ginès J Ariño S Sancho-Bru P Bataller R Pose E. MetALD: clinical aspects, pathophysiology and treatment. JHEP Rep. (2025) 7:101250. doi: 10.1016/j.jhepr.2024.101250 39897615 PMC11782861

[5] Arab JP Díaz LA Rehm J Im G Arrese M Kamath PS . Metabolic dysfunction and alcohol-related liver disease (MetALD): position statement by an expert panel on alcohol-related liver disease. J Hepatol. (2025) 82:744–56. doi: 10.1016/j.jhep.2024.11.028 39608457 PMC12242919

[6] Riley DS Barber MS Kienle GS Aronson JK Von Schoen-Angerer T Tugwell P . CARE guidelines for case reports: explanation and elaboration document. J Clin Epidemiol. (2017) 89:218–35. doi: 10.1016/j.jclinepi.2017.04.026 28529185

[7] Qu H Zhou L Wang J Tang D Zhang Q Shi J. Iron overload is closely associated with metabolic dysfunction-associated fatty liver disease in type 2 diabetes. Obesity. (2025) 33:490–9. doi: 10.1002/oby.24236 39915040 PMC11897857

[8] Britton LJ Subramaniam VN Crawford DH. Iron and non-alcoholic fatty liver disease. World J Gastroenterol. (2016) 22:8112. doi: 10.3748/wjg.v22.i36.8112 27688653 PMC5037080

[9] Zhang L Dai X Wang L Cai J Shen J Shen Y . Iron overload accelerated lipid metabolism disorder and liver injury in rats with non-alcoholic fatty liver disease. Front Nutr. (2022) 9:961892. doi: 10.3389/fnut.2022.961892 36304234 PMC9593083

[10] Chen H. Iron metabolism in non-alcoholic fatty liver disease: a promising therapeutic target. Liver Res. (2022) 6:203–13. doi: 10.1016/j.livres.2022.09.003 39957910 PMC11791839

[11] Cheng Z Chu H Zhu Q Yang L. Ferroptosis in non-alcoholic liver disease: molecular mechanisms and therapeutic implications. Front Nutr. (2023) 10:1090338. doi: 10.3389/fnut.2023.1090338 36992907 PMC10040549

[12] Chen J Li X Ge C Min J Wang F. The multifaceted role of ferroptosis in liver disease. Cell Death Differ. (2022) 29:467–80. doi: 10.1038/s41418-022-00941-0 35075250 PMC8901678

[13] Li C Deng D Jiang Q Shi J Xu L Liu Y. Ferroptosis in NAFLD: insights and the therapeutic potential of exercise. Front Med. (2025) 12:1462145. doi: 10.3389/fmed.2025.1462145 40206477 PMC11979233

